# Supplementary figures and images for: Genomic and phenotypic insights into the expanding phylogenetic landscape of the Cryptococcus genus
Source: PLoS Genet. 2025 Nov 10;21(11):e1011945. doi: 10.1371/journal.pgen.1011945 (PMC12633873; doi:10.1371/journal.pgen.1011945)

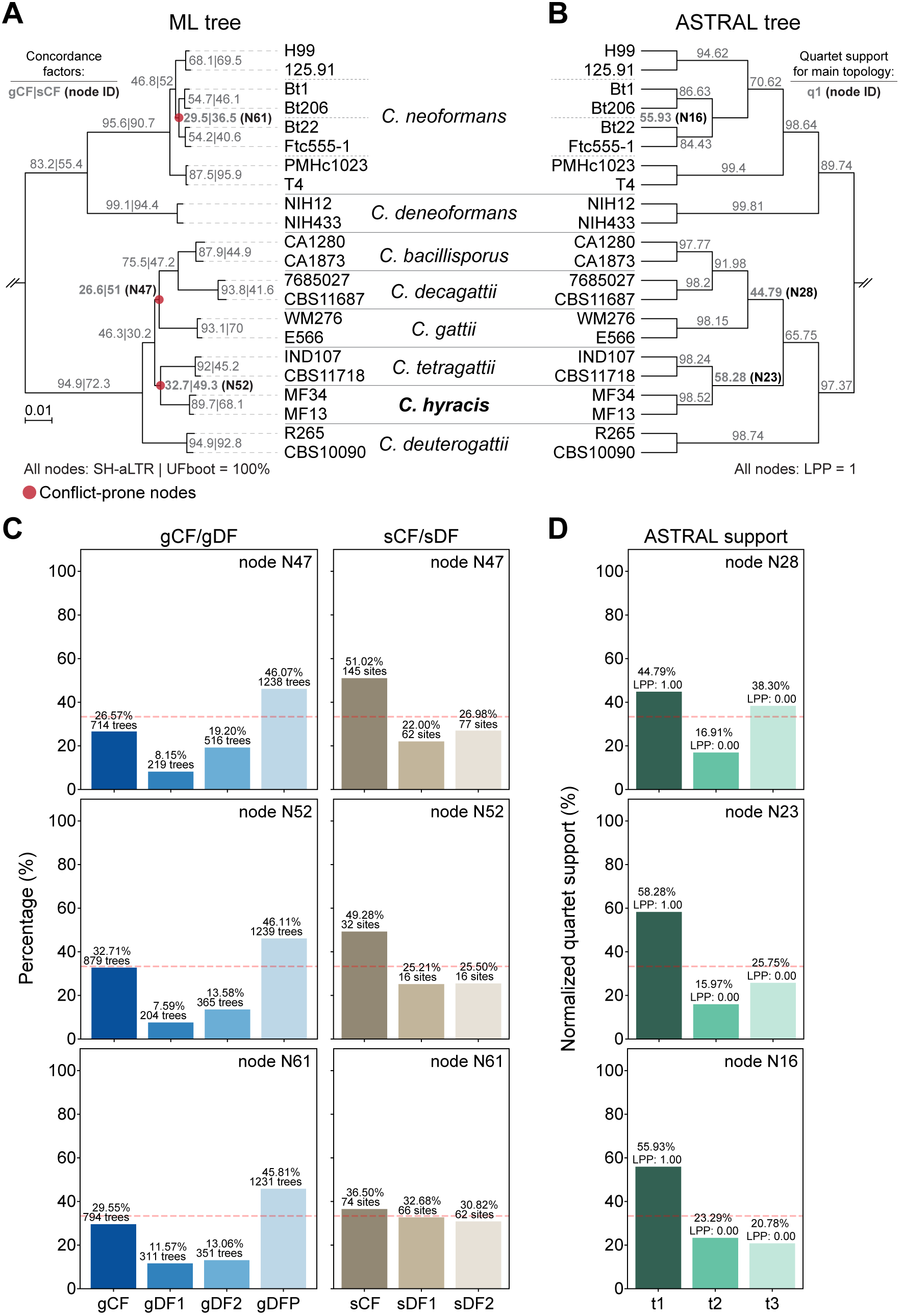

Supplement: S1 Fig — (A) Pruned maximum likelihood (ML) phylogeny (from Fig 1A), showing only clade A species. The tree was inferred from a concatenated alignment of 2,687 single-copy orthologs (SC-OGs). Conflict-prone nodes are marked in red and labeled by node ID. Concordance factors based on gene trees (gCF) and site patterns (sCF) are shown in grey. All nodes are fully supported by SH-aLRT and ultrafast bootstrap (UFboot = 100%). (B) Pruned ASTRAL species tree (from Fig 1B), based on the same SC-OGs. All nodes have local posterior probability (LPP) = 1.0. Quartet support for the main topology is shown in grey. Nodes corresponding to those in panel A are labeled by their ASTRAL node IDs. (C) Gene and site concordance at three conflict-prone nodes (N47, N52, N61). Gene concordance factors (gCF) and discordance factors (gDF1, gDF2, and gDFP) are shown on the left. The main topology is shown in dark blue; alternatives and paraphyly are shown in lighter blue shades. Site concordance (sCF) and discordance (sDF1, sDF2) are shown on the right. The red dashed line at 33.3% represents the expected support under a hard polytomy. (D) ASTRAL quartet support for the corresponding nodes (N28, N23, N16). Bar plots show the normalized frequency of each of the three possible quartet topologies, with the main topology shown in dark green and the two alternatives in lighter shades. Local posterior probabilities (LPP) are indicated above each bar. In all cases, ASTRAL strongly supports the main topology (LPP = 1.0), despite the presence of gene tree discordance in the ML-based analyses (panel C). This highlights how coalescent-based methods can recover consistent species relationships even in the presence of substantial incomplete lineage sorting, which likely underlies the low gCF and high gDFP values observed at these nodes. (TIF) [file pgen.1011945.s001.tif]

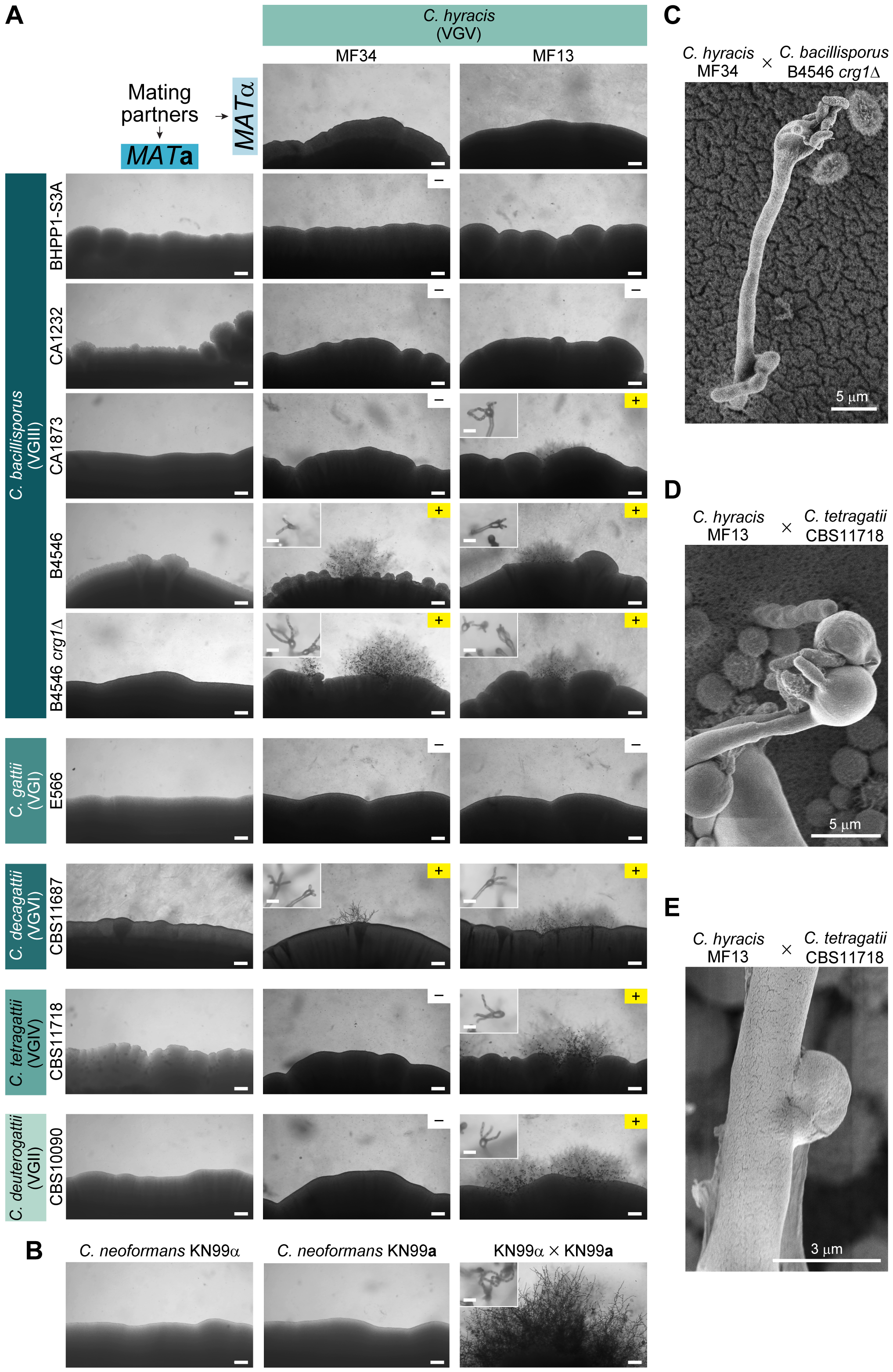

Supplement: S2 Fig — (A) Pairwise mating assays using two C. hyracis MATα strains (MF34 and MF13) crossed with MATa strains from other species in the C. gattii complex. Crosses were performed on V8 agar and examined by light microscopy after 2–3 weeks. Hyphal growth with basidia and spores (inset) indicated successful mating; positive and negative interactions are marked with “+” and “–”, respectively. Strain MF13 generally showed stronger mating responses than MF34. For C. bacillisporus, we tested both the wild-type strain B4546 and a derived crg1Δ mutant. CRG1 encodes a negative regulator of pheromone signaling that acts via G-protein signaling by stimulating GTP hydrolysis to GDP, thereby extinguishing Gα-GTP signaling. Thus, deletion mutants are hypersensitive to mating signals and serve as sensitive indicators of mating potential. Scale bars = 200 μm (10 μm in insets). (B) Positive control cross between C. neoformans MATα (KN99α) and MATa (KN99a) showing robust filamentation and basidia formation. (C–E) Scanning electron microscopy (SEM) of interspecies mating interactions involving C. hyracis. Panels show examples of basidia and basidiospore formation, as well as fused clamp connections, observed in crosses with C. bacillisporus (C) and C. tetragattii (D–E). These structures confirm the ability of C. hyracis to engage in sexual development. (TIF) [file pgen.1011945.s002.tif]

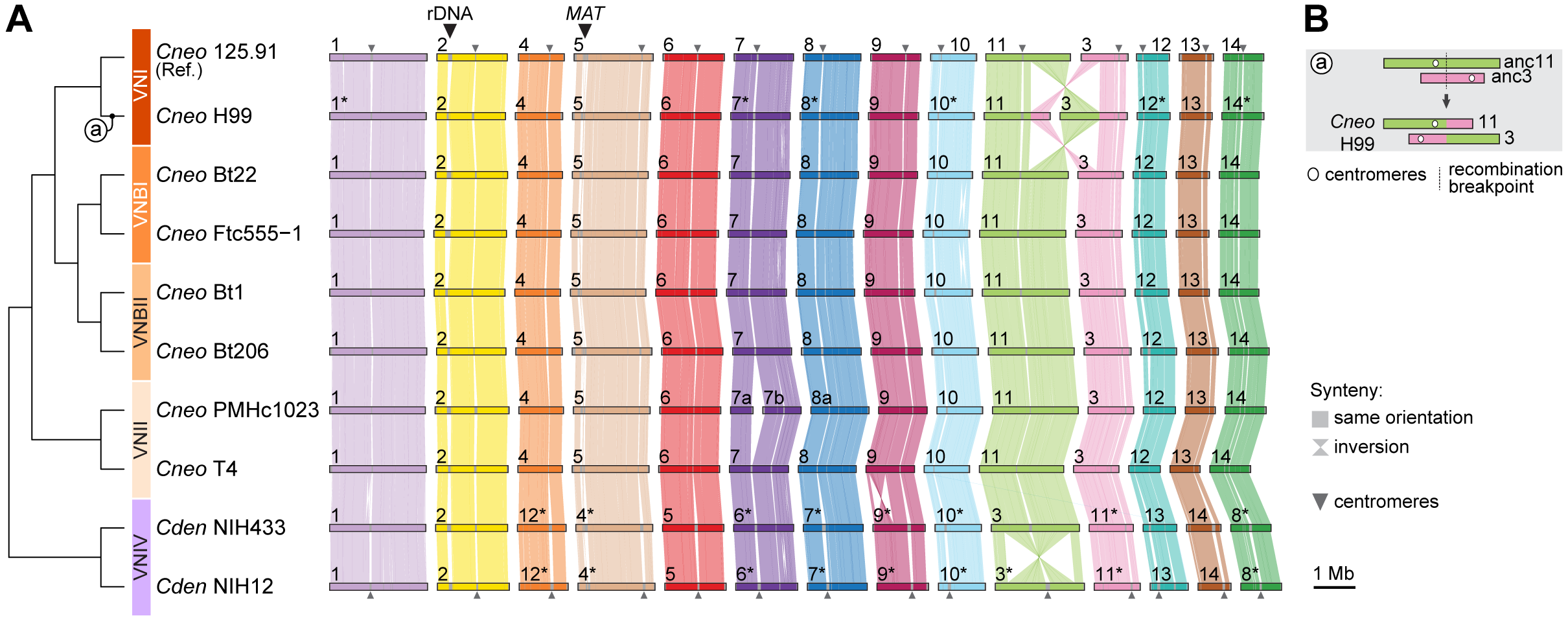

Supplement: S3 Fig — (A) Synteny analysis of 14 chromosomes across multiple C. neoformans strains representing the four major lineages (VNI, VNII, VNBI, VNBII) and two C. deneoformans (VNIV) strains. Chromosomes were ordered to maximize collinearity with the C. neoformans strain 125.91 reference genome, and those inverted relative to their original orientations are indicated with asterisks. Overall synteny is well conserved across C. neoformans strains, except for strain H99 (VNI), which harbors a known translocation between chrs. 3 and 11. C. deneoformans strains are largely syntenic to C. neoformans, except for some large inversions. The locations of the rDNA cluster and MAT locus are indicated. Centromere positions are marked by grey triangles. Rearrangements, including large inversions and translocations, were identified using the ntSynt toolkit and visualized with ntSynt-viz. (B) Schematic of the previously characterized reciprocal translocation in C. neoformans H99 between chrs. 3 and 11. Dashed lines indicate the breakpoint positions, and circles mark centromere locations. This rearrangement distinguishes H99 from other VNI strains. (TIF) [file pgen.1011945.s003.tif]

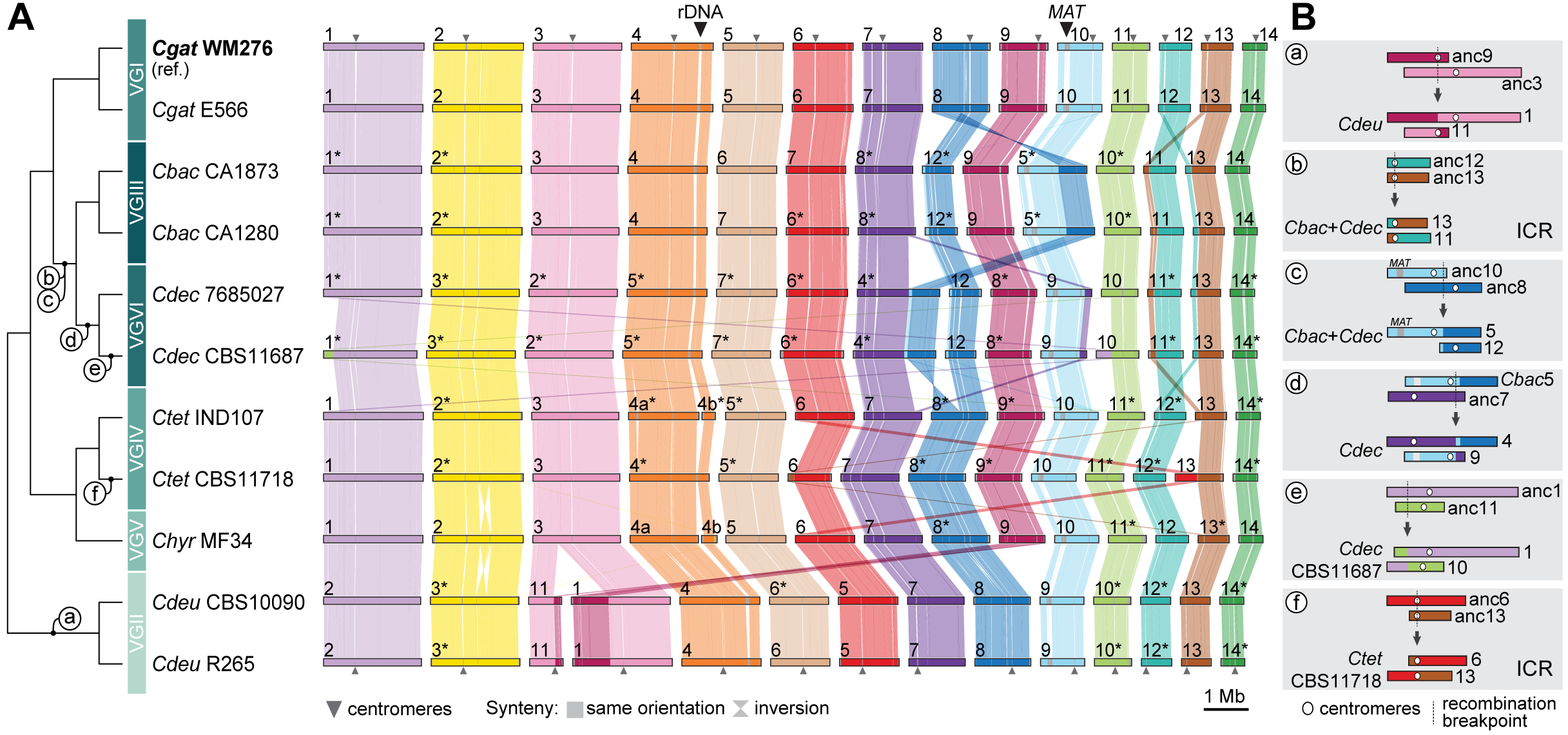

Supplement: S4 Fig — (A) Synteny comparisons between C. gattii strain WM276 (VGI, reference) and representative strains from each of the species within the C. gattii complex. Chromosomes were reordered to maximize collinearity with the reference, and those inverted relative to their original assembly orientations are marked with asterisks. Synteny blocks were identified using the ntSynt toolkit and visualized with ntSynt-viz. Despite broad conservation of synteny, numerous rearrangements are observed across species, including translocations and inversions. The chromosomal locations of the rDNA cluster and the MAT locus are also indicated, as are predicted centromeres (triangles). (B) Schematic representations of selected interspecific chromosomal rearrangements. Colored bars represent putative ancestral chromosomes and their rearranged derivatives in extant species, with breakpoints indicated by dashed lines. Centromeres are shown as circles. Panels highlight key rearrangements across different nodes of the phylogeny, including several likely intercentromeric recombination (ICR) events, such as that shared between C. bacillisporus and C. decagattii (event b) and the derived translocation found in C. tetragattii CBS11718 (event f). (TIF) [file pgen.1011945.s004.tif]

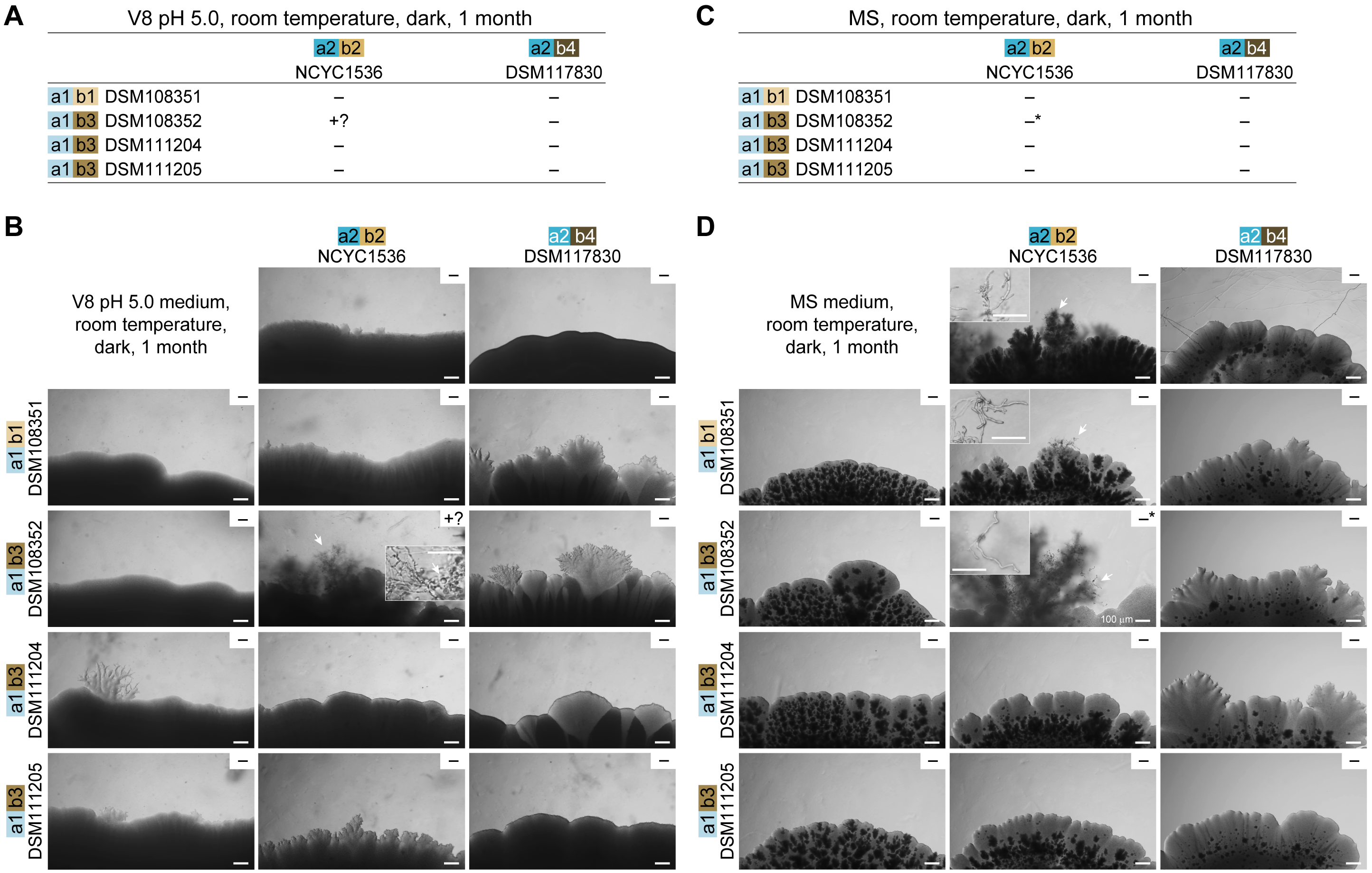

Supplement: S5 Fig — (A) Summary table indicating presence (+?) and absence (–) of hyphal and basidial-like structures. “+?” denotes putative structures consistent with sexual development, though the identity of basidia-like structures could not be definitively confirmed. Mating-type alleles (STE3 and HD) are shown for each strain. (B) Representative light microscopy images from V8 pH 5.0 medium assays. Only the DSM108352 (a1b3) × NCYC1536 (a2b2) cross showed hyphal growth and potential sexual structures embedded in the agar (arrow); the inset highlights a structure resembling a basidium. Scale bars: 200 μm (50 μm for inset). (C) Summary table of mating outcomes on MS medium. (D) Representative light microscopy images from MS medium assays. Pseudohyphal growth, characterized by constricted septa and chain-like elongation, is observed in the solo culture of NCYC1536 and in the DSM108351 × NCYC1536 cross (arrows). True hyphae growth, defined by continuous non-constricted filaments, is observed in the DSM108352 × NCYC1536 cross (arrow), although no apparent basidial-like structures were detected throughout the course of the experiment (marked as “–*”). Scale bars: 200 μm unless otherwise noted (50 μm for insets). (TIF) [file pgen.1011945.s005.tif]

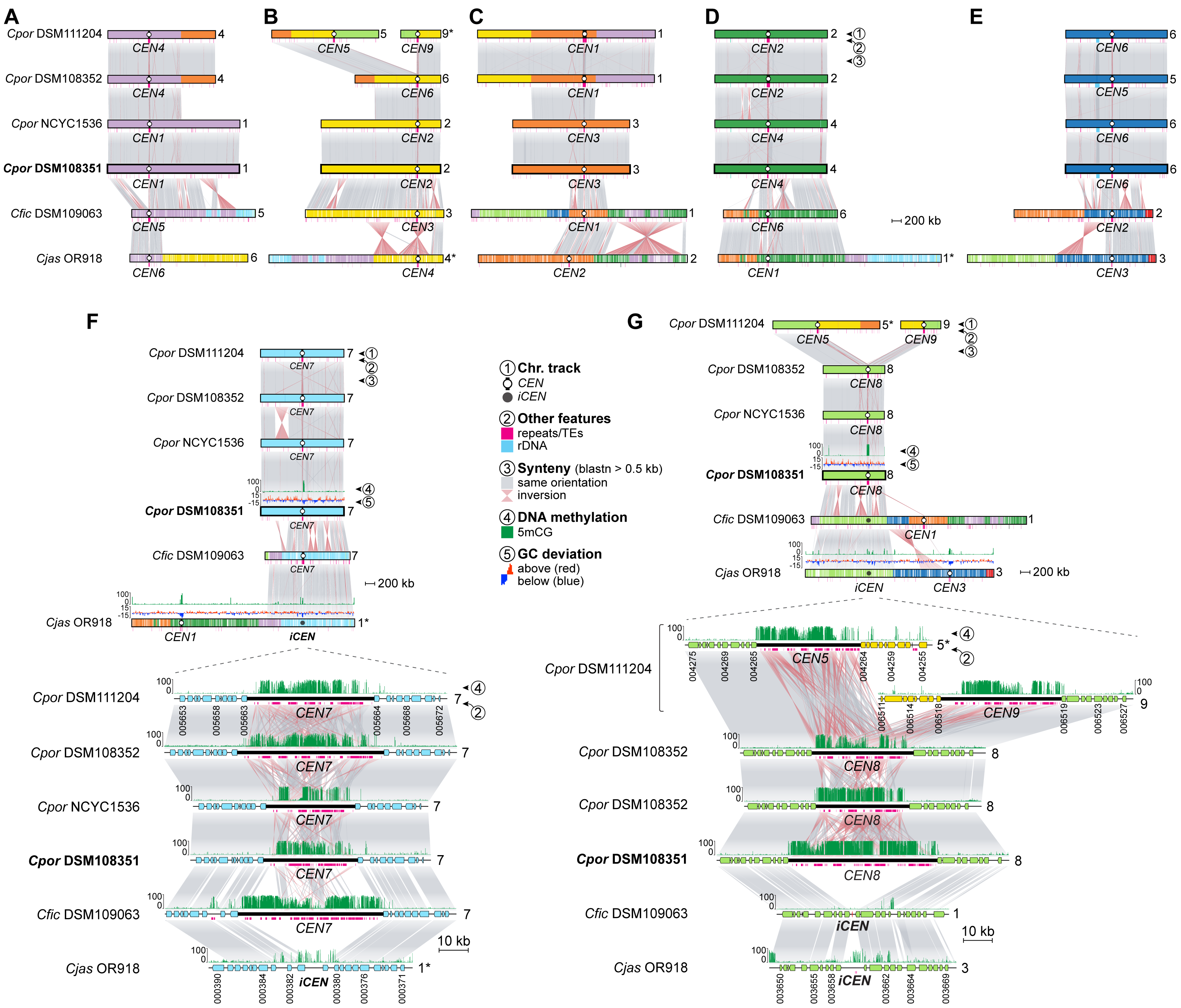

Supplement: S6 Fig — (A–E) Synteny comparisons across conserved centromeric regions in C. porticicola (strains DSM111204, DSM108352, NCYC1536, and DSM108351), C. ficicola (DSM109063), and C. jaspeensis (OR918). Tracks highlight conserved centromere positioning and surrounding synteny for CEN1, CEN2, CEN3, CEN4, and CEN6 of C. porticicola DSM108351 and their corresponding orthologous regions in the other strains and species. (F) Apparent loss of CEN7 in C. jaspeensis OR918. The top panel shows chromosome-scale synteny across strains, highlighting the conserved position of CEN7 in all species except C. jaspeensis, where the orthologous region lacks canonical centromeric signatures and is marked as putative inactivated centromere (iCEN). The bottom panel provides a zoomed-in view of this region with tracks showing synteny, 5mCG DNA methylation, and TE content. Although the centromeric repeat landscape appears disrupted in OR918, residual DNA methylation and partial synteny may indicate a recently inactivated centromere. (G) Apparent centromere loss in both C. ficicola and C. jaspeensis. The top panel shows chromosome-scale synteny, highlighting the position of CEN8 from C. porticicola DSM108351. In strain DSM111204, this region underwent intercentromeric recombination, while in C. ficicola and C. jaspeensis the corresponding region lacks the repeat-rich structure typical of active centromeres. The bottom panel shows a zoomed-in view of this region, indicating that the orthologous loci in DSM109063 and OR918 lack the repeat-rich structure characteristic of active or ancestral centromeres, and only residual 5mCG DNA methylation, consistent with inactivated centromeres (iCEN). (TIF) [file pgen.1011945.s006.tif]

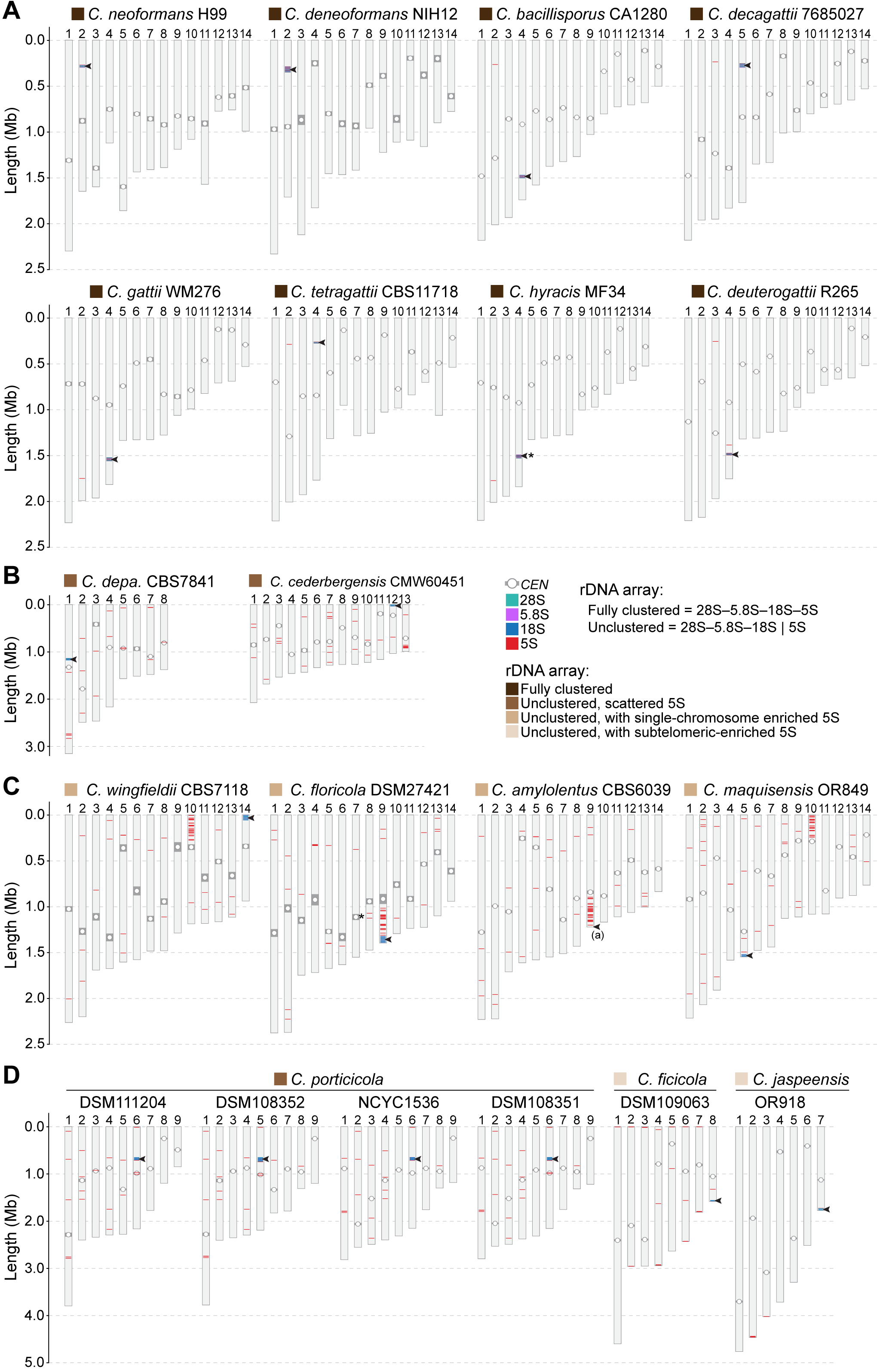

Supplement: S7 Fig — Chromosomal distribution of 28S, 5.8S, 18S, and 5S rRNA genes across all analyzed Cryptococcus strains with chromosome-level assemblies. Arrowheads indicate the position of the core rDNA array, and predicted centromeres are marked with open circles. In C. floricola, chr. 7 is represented as two joined contigs with a gap at CEN7 (marked with an asterisk). The rDNA array (28S–5.8S–18S) is typically clustered on a single chromosome, whereas 5S genes show variable distribution patterns across lineages. (A) Clade A (pathogenic) species exhibit fully clustered rRNA arrays, while saprobic species from other clades (B-D) display unclustered configurations with lineage-specific features: scattered 5S genes (e.g., C. depauperatus and C. porticicola), chromosome-specific enrichment (e.g., C. floricola), or subtelomeric localization (e.g., C. jaspeensis). In C. amylolentus, the 28S–5.8S–18S rDNA cluster is absent from the current assembly owing to unresolved repetitive regions near the end of chr. 9. The inferred position, marked with an arrowhead labeled “(a),” is supported by a few long reads from the original sequencing dataset that extend into the rDNA region. (TIF) [file pgen.1011945.s007.tif]

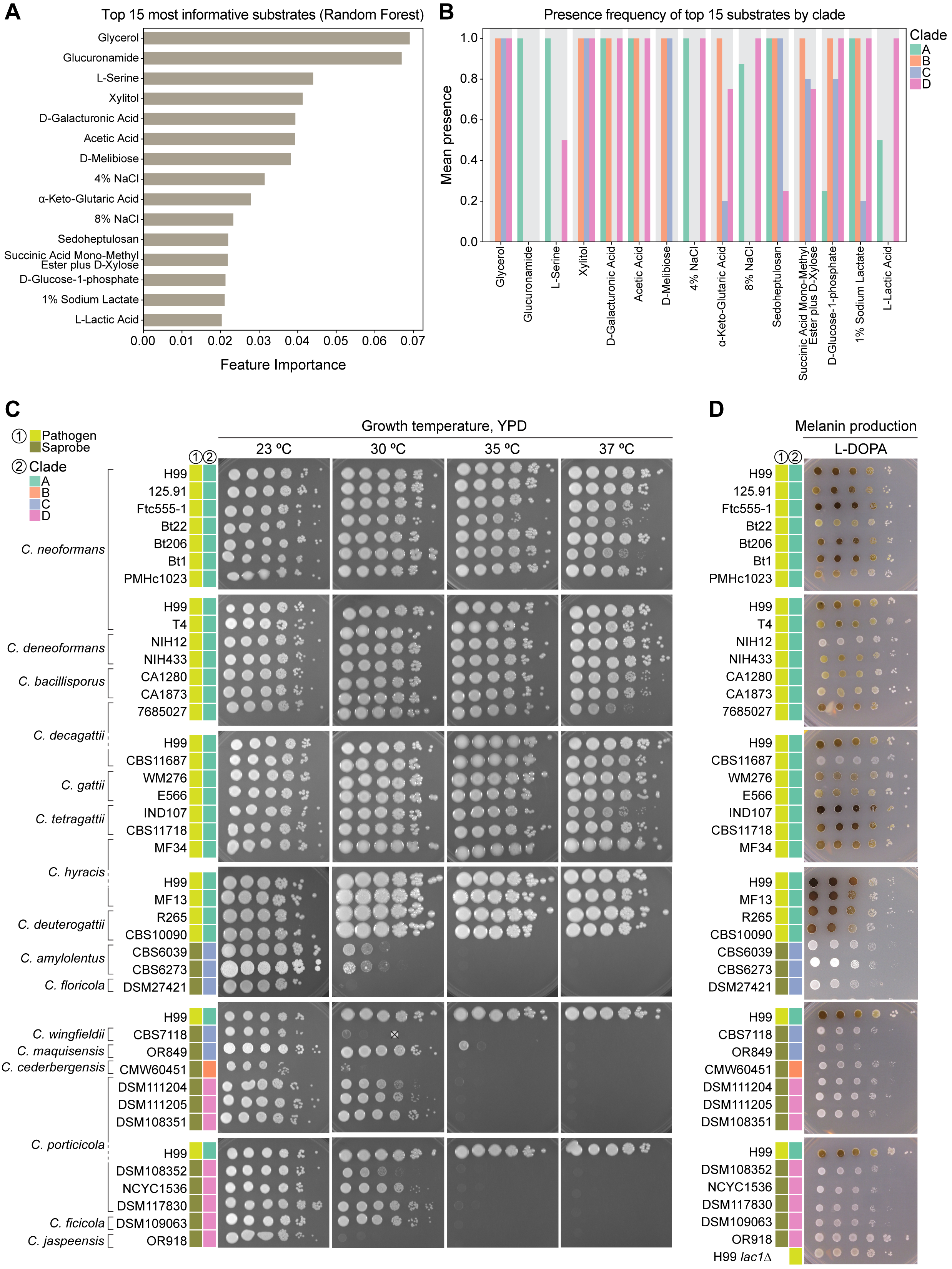

Supplement: S8 Fig — (A) Top 15 phenotypic tests contributing most to clade-level classification based on Random Forest analysis of binarized Biolog data. Substrates are ranked by feature importance in predicting clade membership. (B) Mean presence frequency of the top 15 substrates from panel A across Cryptococcus clades. Some substrates (e.g., glycerol, xylitol, glucuronamide) display clade-specific patterns, with utilization largely restricted to or absent from particular clades, highlighting their potential utility for phenotypic discrimination. (C) Growth of all tested strains at 23°C, 30°C, 35°C, and 37°C on YPD medium for 4 days. Cells were grown for 48 hours in YPD, washed, and spotted in 10-fold dilutions starting from an OD600 of 2.0. Robust growth at 37°C was observed exclusively in clade A strains, consistent with thermotolerance as a pathogenicity-associated trait. No strains from saprobic species in clades B–D showed appreciable growth at this temperature. Among saprobes, C. cederbergensis displayed limited growth above 30°C, whereas C. maquisensis OR849 exhibited weak growth at 35°C (visible in the highest inoculum spot). A colony marked with an “X” represents contamination and should be disregarded. (D) Melanin production on L-DOPA agar plates incubated at room temperature for 5 days. Spotting assay was conducted as described in C. All clade A strains produced visible melanin, while all nonpathogenic strains from clades B–D lacked pigmentation under these conditions. Among pathogens, melanin production was somewhat reduced in C. decagattii CBS11687 and C. deneoformans NIH12 under this growth condition. (TIF) [file pgen.1011945.s008.tif]

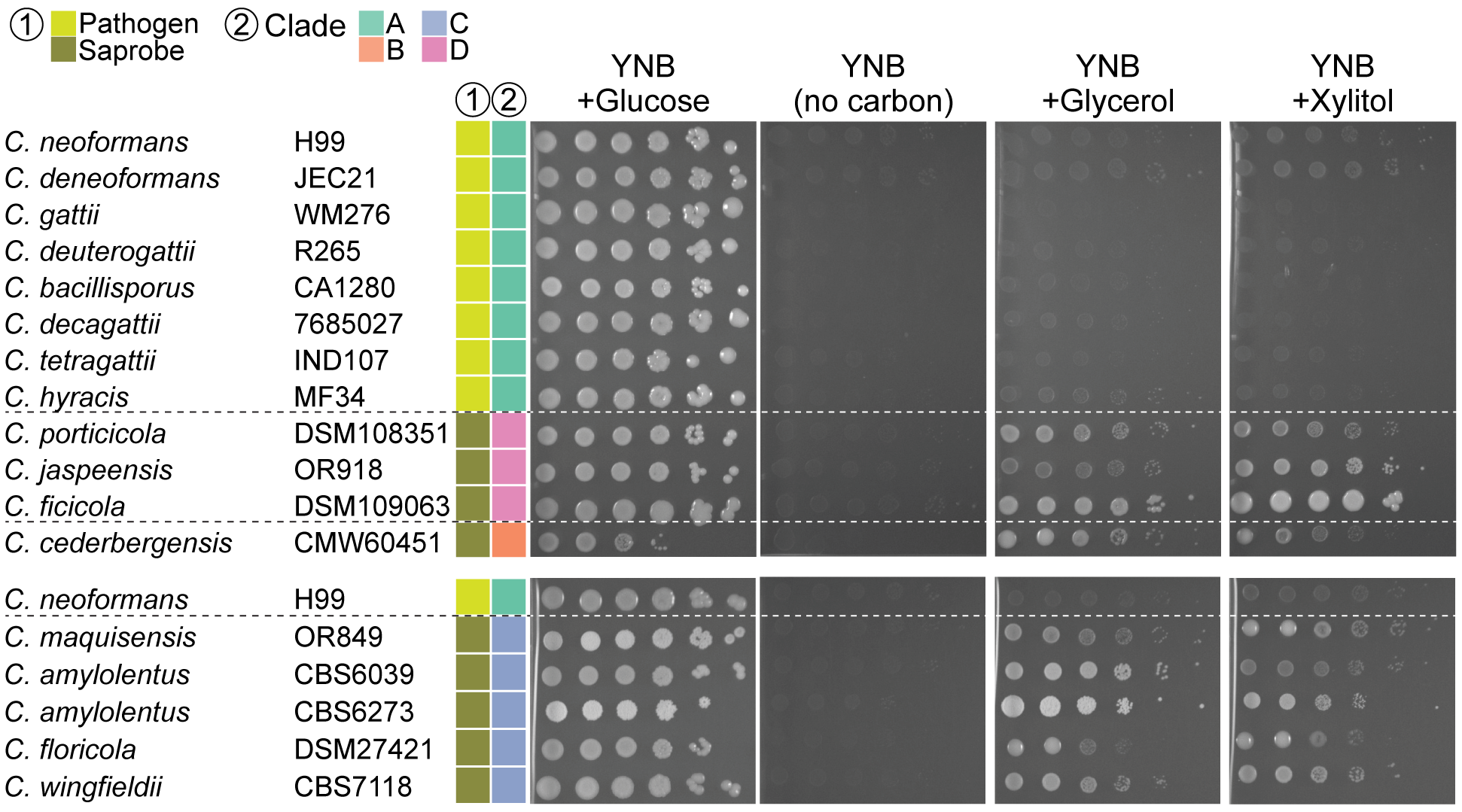

Supplement: S9 Fig — Spot assays testing growth on defined minimal medium (YNB without amino acids) containing glucose, glycerol, or xylitol as the sole carbon source. YNB without added carbon served as a control for potential nutrient carry-over. Cells were pre-grown for 48 h at room temperature (25 ± 1°C) in YNB + 0.1% glucose (carbon-starvation medium), washed, and spotted in 10-fold dilutions starting from an OD600 of 2. Plates were incubated at room temperature for 7 days before imaging. Pathogenic clade A species exhibited little or no growth on glycerol or xylitol, whereas non-pathogenic species (clades B–D) grew well under the same conditions. (TIF) [file pgen.1011945.s009.tif]
