# Supplementary material for: Genomic and phenotypic insights into the expanding phylogenetic landscape of the Cryptococcus genus
Source: PLoS Genet. 2025 Nov 10;21(11):e1011945. doi: 10.1371/journal.pgen.1011945 (PMC12633873; doi:10.1371/journal.pgen.1011945)
Supplement: Attachment — Submitted filename: Response_to_reviewers_R1.pdf [file pgen.1011945.s019.pdf]

Department of Molecular Genetics & Microbiology
Duke University School of Medicine

Response to Reviewers (PGENETICS-D-25-00869)

Reviewer #1's comments:

Reviewer #1: This study reports new genomic characterization of 6 species within the Cryptococcus lineage and places them within a revised species phylogeny for this genus. The authors perform extensive genomic analysis of 17 total Cryptococcus species looking not just at sequence diversity but also at chromosomal rearrangements, karyotype and centromere evolution and rRNA array structure. In addition, they provide phenotypic characterization of mating between strains and species and growth of these species on different substrates. In all, I found this to be a compelling and comprehensive story investigating an important genus of fungi and highlighting interesting themes in genome evolution and the origins of pathogenicity. I think this will be of exceptional interest to those in the Cryptococcus field, but the broad topics covered will likely make this also appealing to a wider audience. That said, I do have a few concerns, questions, and clarifications outlined below:

Response: We thank the reviewer for the thoughtful and encouraging assessment of our work and for recognizing both the breadth and depth of our analyses. We also appreciate the constructive questions and suggestions, which helped us improve the clarity and balance of the Discussion without altering our conclusions. In revising the manuscript, we made targeted text edits to clarify our operational framework for delimiting species and how to integrate multiple lines of evidence. Below, we respond point-by-point to each comment and indicate the corresponding revisions. Please note that line numbers refer to the clean version of the revised text (without tracked changes).

Major Comments

-The authors discuss species concepts/speciation throughout, but I came away from this paper confused about how they would actually like me to interpret what a "species" is and how to define species boundaries. They start to lay out how it can be powerful to take a holistic approach incorporating many different types of data in these decisions including: nucleotide/sequence diversity, chromosomal diversity, rDNA structure, mating, and metabolism. But then they launch into descriptions of the data and I am missing how they propose to incorporate all of this data into the decision of where to draw species boundaries? I don't disagree with the distinctions they draw in the paper, but among their test cases are: C. hyracis vs. C. tetragattii, which they call separate species while having high(er) sequence divergence, low chromosomal structure/rDNA divergence, similar metabolism, and seem to mate (though knowing productivity seems very important here!) and the C. porticicola strains, which they group together as a single species, that have low sequence divergence, low karyotypic diversity, similar metabolism and largely don't mate with each other. These only really seem to differ in sequence divergence and mating capacity. Does this suggest that the authors think sequence level diversity is the most useful in defining species boundaries?

Response: We appreciate the reviewer's attention to this point and agree that our original Discussion did not state explicitly how we integrate the different evidence types. We have now added a new paragraph (**lines 758–778**) that defines our taxogenomic framework and the weighting of evidence: phylogenomic monophyly and genome-wide divergence (empirically

calibrated, not fixed cutoffs) are the primary indicators of long-term evolutionary independence; chromosomal structure, sexual compatibility, and ecology provide secondary support and can lag or lead in time relative to sequence divergence. We then apply this logic explicitly to the two cases raised by the reviewer:

- *C. hyracis* vs. *C. tetragattii*. Despite largely collinear karyotypes and formation of sexual structures in interspecific pairings, genome-wide divergence and monophyly support species-level separation. The observed ANI and dDDH values fall below intraspecific values for *C. neoformans* and even below those for the closest recognized interspecific pair in our dataset (*C. bacillisporus*–*C. decagattii*), which we point out in the Discussion.

– *C. porticicola* strains. All strains share >99% ANI and a conserved 9-chromosome karyotype but show strain-specific rearrangements and low mating efficiency. Because progeny viability could not be assessed and some strains carry private rearrangements, we primarily rely on genomic cohesion. We emphasize that genome-wide signals of recombination (reticulate network, significant PHI test, LD decay with LD50 ≈ 13.9 kb) support their classification as a single recombining species undergoing population-level diversification.

Again, I have no real issue with the phylogeny they propose and I liked how they presented data on the many different axes of genomic/biological diversity that might contribute to speciation and reproductive isolation. But there's both inter- and intra-species variation across all of these axes. I think my confusion here would largely be addressed just by a clearer synthesis of the data as a whole in the discussion, specifically with regard to how to go about defining what a species is and how the authors are integrating the datasets they collected here (or not).

Response: We thank the reviewer for requesting a clearer synthesis. We have added a paragraph that states our operational, integrative framework and the weighting of evidence (genome-wide divergence + phylogenomic monophyly as primary; chromosomal, reproductive, and ecological data as secondary, potentially lagging/leading). We also note (**lines 816–817**) that this framework is iterative and as additional isolates and genomes are obtained, including opposite mating types and broader geographic/ecological sampling, the intraspecific floor and interspecific ceiling of genomic similarity can be recalibrated, and species assignments can be revisited on a clade-specific basis.

I do also think analysis of the viability of progeny in the matings they show would add significantly to this manuscript and to their discussion of speciation.

Response: We agree that assessing progeny viability would provide complementary evidence of post-zygotic isolation and strengthen the application of the biological species concept (BSC) to the presented model. Two practical constraints currently limit such assays: (i) all available *C. hyracis* isolates are *MAT*α, precluding conspecific (α × **a**) controls to benchmark viability; and (ii) in *C. porticicola*, mating is rare/low-efficiency in the only compatible pair, which also differs by two reciprocal translocations expected to reduce meiotic viability even within species. As noted in the Discussion, our taxogenomic framework prioritizes genome-wide divergence and phylogenetic distinctness when reproductive data are incomplete or variable; moreover, we provide independent genomic evidence for recombination in *C. porticicola* (reticulate relationships, significant PHI test, and LD decay). Although these parameters do not fully replace the BSC, some genomic features can serve as proxies for it in the absence of direct evidence based on reproductive compatibility.

We agree that obtaining *MAT***a** isolates of *C. hyracis* and expanding reproductive assays across the *C. gattii* complex in future studies will also help refine clade-specific genomic benchmarks, and we state this in the revised Results (**lines 304–306**) and Discussion (**lines 812–817**) sections. One challenge is that progeny recovery in the *C. gattii* complex is technically more difficult compared to *C. neoformans*/*C. deneoformans* because spore chains are short and fragile. Robust viability assays will also require analysis of (i) conspecific α × **a** crosses between strains with closely matched karyotypes (i.e., lacking large translocations) and (ii) parallel interspecific pairings likewise selected to minimize major chromosomal differences. Accordingly, strain selection will need to be guided by telomere-to-telomere (T2T) assemblies that can reveal potential intercentromeric rearrangements that are not detectable with short-read assemblies.

- It seems premature to make assumptions about the metabolic profiles of the species based solely on the biolog data without validating through other methods. And in general, the phenotyping section of this paper seemed a bit preliminary compared to the rest of the manuscript. It would be nice if the authors could validate growth/no growth phenotypes (or perform more nuanced growth assessments) by another method for at least the substrates pulled out by the random forest model. Maybe they already did this, but I found the methods and discussion of the auxanographic tube tests to be challenging to parse and this was not mentioned anywhere in the main text.

Response: We thank the reviewer for this important point. We agree that Biolog data alone should be interpreted with caution, and have substantially expanded the Methods section (**lines 1235–1287**) to clarify the validation framework and complementary assays used. In this study, Biolog assays were not analyzed in isolation but benchmarked against standard assimilation tests from classical yeast taxonomy (tube and custom microplate assays) and publicly available sources of physiological tests (e.g., CBS-WI, PYCC databases; and literature, such as *The Yeasts: A Taxonomic Study*). This benchmarking covered 12 of 17 recognized species (71%), including both pathogenic and saprobic lineages, and validated 46 of 153 substrates tested (30%).

In addition, two informative substrates identified by the Random Forest analysis (glycerol and xylitol) were re-tested through independent spot assays on defined YNB medium at 25 ± 1°C. These confirmatory assays qualitatively corroborated the Biolog results, showing robust growth of saprobic species (clades B, C, and D) but limited or no growth of pathogenic clade A species when each substrate was provided as the sole carbon source. These results are now described in **lines 510–515** of the revised Results section and illustrated in the new Supplementary Figure S9.

We also note that, for the formal descriptions of the novel species, assimilation tests were performed in liquid and microplate formats following the protocol of Kurtzman et al. (2011), providing additional cross-validation for selected substrates. This is now detailed in the revised Methods section (**lines 1289–1301**). Thus, we clarify the integrated workflow combining (i) Biolog assays, optimized for broad comparative profiling, and (ii) traditional growth assays, used for species descriptions and cross-validation.

I was also particularly interested in the glycerol result. Namely, that clade A does not grow on glycerol. There are several published papers (PMCIDs: PMC2730461, PMC3110414, PMC8092316) which all show fairly robust growth for the C. neoformans reference strain (H99) on glycerol. I'm curious how the authors interpret this discrepancy?

Response: We thank the reviewer for this careful observation and for highlighting the relevant studies reporting *C. neoformans* growth on glycerol. We carefully reviewed the referenced papers

and note that their experimental setups differ substantially from the defined carbon-assimilation assays used in our study. Both PMC3110414 and PMC8092316 explicitly describe pre-culturing cells in rich YPD medium at 30°C prior to spotting onto either YP + glycerol or YNB + glycerol plates. In PMC2730461, pre-culture conditions were not detailed, but given that all other assays in that study used overnight YPD cultures, it is likely that the same procedure was followed. Such pre-growth in complex media and at 30°C differs fundamentally from carbon-assimilation assays, where cells are carbon-starved before testing. Residual intracellular metabolites from rich media or stored compounds (e.g., trehalose; Ngamskulrungroj et al., 2009, PMID 19651856) can transiently sustain growth even when the tested substrate is not efficiently metabolized.

By contrast, our experiments followed standardized carbon-assimilation protocols (Biolog Phenotype MicroArrays and traditional methods in *The Yeasts: A Taxonomic Study*), in which cells are transferred to defined YNB minimal medium containing a single carbon source. To minimize nutrient carry-over, inocula were pre-grown for 48 h in YNB + 0.1% glucose (carbon-starvation medium) before testing. Under these conditions at 25 ± 1°C, pathogenic clade A species, including *C. neoformans* H99, failed to grow efficiently on glycerol as the sole carbon source, whereas saprobic species from clades B–D displayed robust growth. As mentioned above, these results were further confirmed by independent spot assays on YNB media supplemented with glucose, glycerol, or xylitol (new Fig. S9).

We therefore interpret the discrepancy between our findings and prior reports as arising primarily from differences in inoculum preparation, nutrient context, and possibly temperature. While *C. neoformans* H99 can apparently survive or grow on glycerol at higher temperatures (30 and 37°C) when residual nutrients are available (as reported by others), our data indicate that clade A species do not efficiently assimilate glycerol as a sole carbon source under nutrient-limited conditions at ~25°C. We now highlight this potential temperature dependency explicitly in the Discussion (**lines 976–984**).

Lastly, on this point, I'm curious if there are any logical explanations for how the most informative growth features correlate with distinct environments. For instance, are natural sources of xylitol correlated with the isolation sites of species that grow on it? Again it might be useful to have another complementary experiment for some of these metabolic traits that both confirms the biolog data but also potentially gives a more nuanced view of differences in growth. I don't think this is essential, but it might help to strengthen the argument that ecological adaptation underlies some of the discordance between the metabolism/phenotypic data and the phylogenetics.

Response: We thank the reviewer for this thoughtful comment. We have expanded the Discussion (**lines 958–975**) to address potential ecological correlates of the observed metabolic differences. In particular, we discuss the clade-level pattern of glycerol and xylitol assimilation mentioned above, which may reflect adaptation to polyol-enriched environments such as decaying wood and bark-beetle–associated habitats. Xylitol, derived from xylose in plant hemicellulose, occurs in plant saps, fruits, and decaying wood, and is commonly metabolized by yeasts inhabiting such substrates (e.g., Cadete et al., PLOS One, 2012; PMID: 22912807). Glycerol and related polyols are also present in bark-beetle–associated habitats, where they accumulate in insect tissues as cryoprotectants and metabolic intermediates (e.g., Kostál et al., J Insect Physiol., 2007; PMID: 17481650). The broader capacity for polyol assimilation among species of clades B, C, and D, many of which are plant- or insect-associated taxa, may therefore represent ecological adaptation to these niches. Although preliminary, we hope these findings will motivate future studies exploring links between metabolic flexibility and environmental specialization across *Cryptococcus*.

Minor Comments/Questions:

lines 156-157 and throughout: How were C. neoformans strains chosen? Do they incorporate the maximum diversity available in strains of this species? This could be useful information to provide. Also seems important to consider the relative relatedness given the authors use of these strains to determine maximum intraspecies divergence--e.g. how much would the AAI and ANI and dDDH data change with different C. neoformans strain selections? I doubt it would change any interpretations but could be useful to discuss or compare.

Response: We thank the reviewer for raising this point. The *C. neoformans* strains analyzed were selected to encompass the full known within-species diversity based on the population structure defined by Desjardins et al. (Genome Res. 2017, PMID: 28611159). We included two representative isolates from each of the four major VN lineages (VNI, VNII, VNBI, VNBII). Within each lineage, strain selection was guided by (i) availability of complete, chromosome-level genome assemblies, (ii) inclusion of opposite mating types where possible (VNII currently lacks *MAT***a** isolates), and (iii) representation of divergent sublineages following Desjardins et al. For example, within VNI we included H99 (VNIb) and 125.91 (VNIa), which belong to distinct subclades (Desjardins, Suppl. Fig. 1). Because genome-wide nucleotide diversity is low within VN lineages compared to the deeper divergence among lineages, substituting an alternative VNII isolate would not materially alter inter-lineage ANI, AAI, or dDDH estimates or affect any conclusions or thresholds. We now explicitly state in **lines 167–171** that:

"For C. neoformans, we further incorporated two representatives from each of the major lineages (VNI, VNBI, VNBII, VNII) to reflect known within-species diversity. Strains were chosen based on the availability of complete genome assemblies, inclusion of opposite mating types where possible, and representation of divergent subclades within each lineage as previously defined [33]".

-lines 413-414: It was very unclear to me until I got to the methods that the methylation analysis used in defining centromere locations was performed by the authors? Was all of the methylation data presented from this study? It would be useful to clarify what data was collected in this study vs. what came from published/other datasets (if anything).

Response: We thank the reviewer for this comment. All 5mCG DNA methylation analyses presented here were performed from raw Oxford Nanopore sequencing data generated either in this study or in our previous work. Specifically, Nanopore data were newly generated for *C. porticicola* strains DSM108352 and DSM111204, whereas previously produced Nanopore datasets were reanalyzed for *C. jaspeensis* OR918 (Coelho, David-Palma et al., PLOS Biol. 2024; PMID: 38843310) and for *C. porticicola* DSM108351, NCYC1536, and *C. ficicola* DSM109063 (Coelho, David-Palma et al., PLOS Biol. 2025; PMID: 41042803). For older datasets originally available only as FAST5 files, raw signals were first converted to POD5 prior to Dorado basecalling to ensure consistency across all genomes analyzed, as is now explicitly stated in the Methods (**lines 1175–1176**). Thus, while some of the underlying read data were published previously, all methylation calls, comparative analyses, and visualizations are new and were generated as part of the present work. We have clarified this distinction in the Results (**lines 428–430**), were we state:

"DNA methylation calls were generated from Oxford Nanopore sequencing data obtained either in this work or from previously published datasets [30, 32]."

-lines 915-917: I find the conservation of melanin biosynthesis genes across species very intriguing. Are there also transporters that are required for taking up melanin pre-cursors (e.g. L-DOPA)? Are these melanization genes/laccases used for other purposes? Are all of these predicted genes functional (i.e. have any been pseudogenized)?

Response: We appreciate the reviewer's questions. We focused on contrasting the conservation and copy number of laccase genes, which encode the core enzymatic drivers of melanization in pathogenic *Cryptococcus* species, and on relating these patterns to pigment formation on two standard assays: bird seed agar and L-DOPA medium. Bird seed agar contains natural phenolic precursors (e.g., caffeic and related catechols) that are oxidized by cryptococcal phenoloxidase (laccase), producing the diagnostic brown/black pigmentation. It is therefore a long-standing, selective differential medium for assessing phenoloxidase-dependent melanization in *Cryptococcus*. In contrast, L-DOPA medium provides a defined single precursor to assess laccase-dependent melanin formation independent of the variability inherent to plant extracts. Together, these assays are complementary: one more ecologically realistic and selective (bird seed), and the other chemically defined (L-DOPA). The rationale for using these two media has now been added in the revised Methods section (**lines 1313–1317**).

We did not perform a *de novo*, genome-wide re-annotation of genes previously implicated in melanization, as this was addressed in our earlier work with a smaller yet representative *Cryptococcus* species panel (Coelho, David-Palma et al., PLOS Biol. 2024; PMID: 38843310). However, following the reviewer's suggestion, we re-inspected all laccase homologs in detail and did not observe obvious pseudogenization (e.g., gross truncations or frameshifts) except for one laccase-like copy in *C. wingfieldii* that is markedly shorter and thus potentially nonfunctional. Accordingly, we revised the *C. wingfieldii* laccase copy number from 4 to 3 and added a note in the Fig. 7D legend indicating the presence of this truncated ORF (**lines 1901–1902**). Because we currently lack expression data, we refrain from asserting gene functionality beyond the presence of apparently intact ORFs.

Regarding precursor uptake and transporters, classical physiological work in *C. neoformans* (Polacheck et al., J. Bacteriol. 1982; PMID: 6804439) seems to demonstrate energy-dependent (azide-sensitive) active uptake of L-DOPA and catecholamines and reported high-affinity kinetics for DOPA, dopamine, norepinephrine, and epinephrine. This constitutes functional evidence for a dedicated transport system, although the responsible genes were not identified in that pre-genomics study. Subsequent genetic work has linked catechol uptake and oxidation to oxidative-stress protection, reinforcing the role of transport processes in melanization. However, to our knowledge, the specific genes or transporters mediating this uptake remain unidentified, though they may involve large neutral or aromatic amino acid transporter families, which warrants further investigation.

Finally, we note that cryptococcal laccase (phenoloxidase) oxidizes a broad range of phenolic compounds, including catechols and polyphenols, consistent with broader ecological functions such as the turnover of plant-derived substrates and environmental detoxification, in addition to host-associated virulence. This likely explains the conservation and occasional expansion of laccase homologs in nonpathogenic lineages. We also highlight in the revised Discussion that the expanded laccase copy number observed in Clade C could are probably linked to non-virulence roles and could represent preadaptive traits later co-opted for pathogenicity.

These points are now reflected in the revised Discussion section, **lines 985–1000**.

-Figure S7C: It appears from the figure that C. amylolentus has no core rDNA array and no 28S-5.8S-18S sequences anywhere in the genome? This doesn't seem correct?

Response: We thank the reviewer for catching this. *C. amylolentus* does contain a complete 28S–5.8S–18S rDNA array, but this region is not represented in the current assembly due to its high repetitiveness and the lower-quality read data from older sequencing technology used to generate that assembly in 2016. The absence of the rDNA array in S7C Fig. therefore reflects an incomplete assembly at the end of chr. 9. We re-mapped the original long-read data and confirmed that a few reads extend into the rDNA cluster, consistent with its presence but with insufficient coverage to support proper assembly. We now indicate this caveat in the figure legend and have added an arrowhead (labeled "a") to mark the inferred position of the core rDNA cluster for consistency with other species.

The figure legend now reads:

"In C. amylolentus, the 28S–5.8S–18S rDNA cluster is absent from the current assembly owing to unresolved repetitive regions near the end of chr. 9. The inferred position, marked with an arrowhead labeled "(a)," is supported by a few long reads from the original sequencing dataset that extend into the rDNA region."

We also wish to mention that additional evidence from more recent, higher-quality Nanopore long-read sequencing and reassembly of *C. amylolentus* confirms that the 28S–5.8S–18S rDNA array is indeed located near the end of chr. 9, similar to that observed in *C. floricola* (see figure below). As this updated assembly remains unpublished and is part of an independent study (in preparation), we have chosen not to include it in the current manuscript.

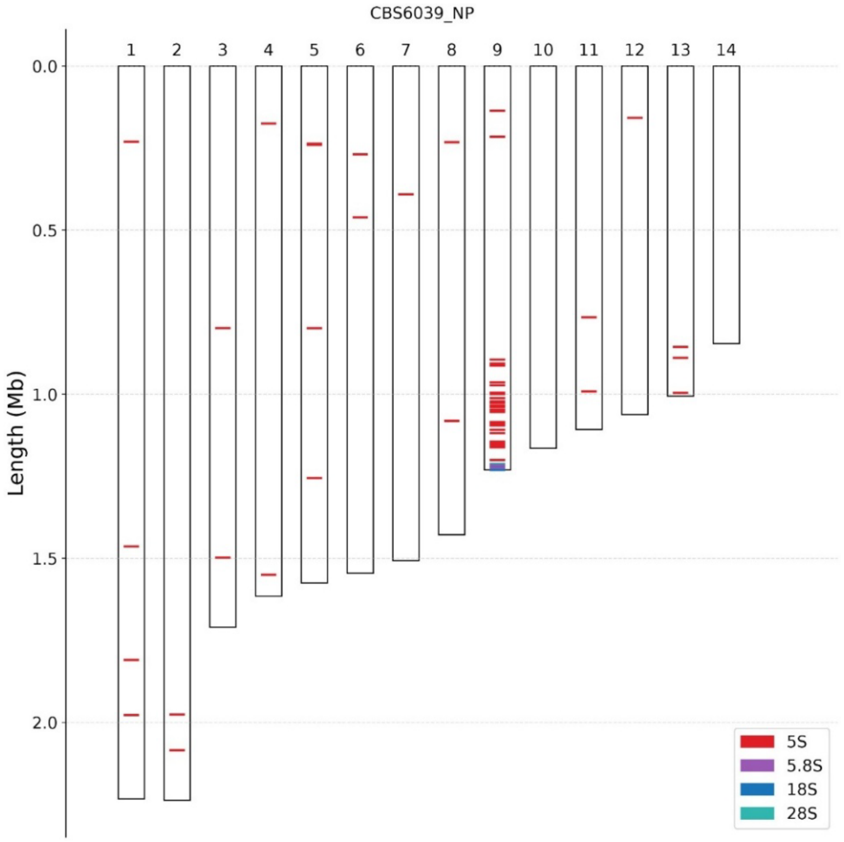

Reviewer #2's comments:

Reviewer #2: The manuscript "Genomic and phenotypic insights into the expanding phylogenetic landscape of the Cryptococcus genus" by Coelho et al. presents an in depth examination of genomic and phenotypic diversity within the important fungal genus Cryptococcus. This includes the formal description of novel species as well as numerous intriguing observations about various facets of genome evolution in this group, much of which has important implications for the virulence of many of the strains. The manuscript is in excellent shape and can be published as is in my opinion. The authors have done a fantastic job with all of the analyses, presentations of results, and discussions. I'm particularly pleased to see the ANI analysis applied to species concepts here as this is something that is sorely needed in fungal taxonomy/systematics. My only overall critique is that the authors should tone down their language with respect to "centromere inactivation". Without data showing the location of the centromeric histones, the results, while compelling, are inconclusive. In particular I feel that lines 49/50 in the Abstract is too assertive given the data. Beyond this I only have minor (mostly pedantic) points that the authors could consider for a revised version, if they choose.

Response: We thank the reviewer for the very positive and encouraging assessment of our manuscript and for the constructive suggestions provided. We appreciate the recognition of the analytical depth of our study and the potential value of the ANI framework for fungal species delimitation. In response to the reviewer's main comment, we have carefully revised the language referring to "centromere inactivation" throughout the manuscript, including the Abstract, to ensure a more cautious phrasing aligned with the available evidence. Below, we address each of the reviewer's additional comments in detail and outline the corresponding revisions made to the manuscript. Please note that line numbers refer to the revised clean version of the text (without tracked changes).

I like the historical context in the intro, maybe add to the opening paragraph that many species formerly called Cryptococcus have been delimitated to new genera.

Response: We thank the reviewer for this helpful suggestion. We have revised the Introduction to briefly note that many yeasts historically classified as *Cryptococcus* have been reassigned to other genera within the Tremellomycetes following molecular and phylogenetic revisions. We agree this addition provides important taxonomic context and acknowledges the major systematic updates that clarified the boundaries of *Cryptococcus* sensu stricto. The revised sentence now reads in **lines 97–101**:

Early molecular studies revealed that the genus Cryptococcus is polyphyletic, encompassing distantly related yeasts within the class Tremellomycetes [8, 9]. Subsequent phylogenetic revisions resolved this taxonomic heterogeneity, transferring taxa unrelated to the pathogenic lineage to other genera such as Naganishia, Papiliotrema, and Cutaneotrichosporon [10, 11].

Figure 1A has C. hyracis as a pathogen, but this is hypothetical, correct?

Response: We thank the reviewer for pointing this out. *Cryptococcus hyracis* (formerly *C. gattii* lineage VGV) has not been associated with any confirmed human infections to date (Farrer et al., mBio 2019; PMID: 31719178). However, it exhibits thermotolerance and other traits (e.g., melanin production) that resemble those of pathogenic species in clade A, suggesting a potential for pathogenicity. To clarify this distinction, we have revised Fig. 1A by adding an asterisk next to *C.*

hyracis strains to indicate that this species has not yet been associated with human infections, and updated the figure legend accordingly. In the study by June Kwon-Chung and Mat Fisher cited above, they found that this species can cause limited pulmonary infections in mice without dissemination to the CNS. Because the majority of *Cryptococcus* clinical isolates are derived from CSF from patients with CNS infections, future studies may need to focus on pulmonary samples (e.g., sputum or BAL) from patients in endemic regions in sub-Saharan Africa to ascertain whether *C. hyracis* may cause a different pattern of infection (primarily pulmonary rather than CNS).

Is Figure 1C needed in a main figure? This type of workflow is more or less standard now for phylogenomic analyses, unless I am missing some novel approach?

Response: We thank the reviewer for this comment. We agree that the overall structure of the workflow in Fig. 1C follows current phylogenomic standards. However, we think that retaining this schematic in the main figure provides helpful context for interpreting the support metrics (gCF, sCF, and ASTRAL quartet support) and for following the sequential filtering steps applied to our dataset without needing to refer to the Methods section. In particular, our workflow includes several additional quality-control filters compared to other *Cryptococcus* studies. We retained only proteins that were ≥100 amino acids in length, required that orthologs be at least 50% of the *C. neoformans* H99 protein length, and further filtered alignments to retain only those with >30% parsimony-informative sites using PhyKIT to improve signal quality. We therefore consider inclusion of this schematic informative for readers wishing to reproduce or extend these analyses.

For Figure 1D & E are the alternative topologies visualized somewhere? Same Question for Figure S1.

Response: We thank the reviewer for this point. The alternative topologies corresponding to the conflict-prone nodes in the ML and ASTRAL trees (e.g., N43/N32 in Fig. 1; N4/N28, N52/N23, and N61/N16 in Fig. S1) are not shown as separate tree diagrams, as neither IQ-TREE nor ASTRAL directly generates graphical representations of the alternative quartet resolutions. Instead, these are summarized quantitatively through the gene and site concordance factors (gCF and sCF) in Fig. 1D and through the quartet frequency distributions in Fig. 1E, which together capture the relative support for each of the three possible topological resolutions. Because these nodes involve large multi-taxon partitions rather than simple quartets, direct visualization of all alternative topologies would add substantial complexity without additional interpretive value. We therefore consider that these summary metrics effectively convey the same information that separate visualizations would provide.

To facilitate transparency and reproducibility, we have now included in S2 File the complete set of ML gene trees (trimal_pergene.treefile), the version with branches collapsed below 70% ultrafast bootstrap support (trimal_pergene.BS70.treefile) that served as input to ASTRAL, and the corresponding ASTRAL quartet annotation file containing topology partitions and frequency data (trimal_pergene.t16.freqquad.csv). This is now indicated in the Methods section (**lines 1114–1116**).

Maybe mention that C. neoformans and C. deneoformans can hybridize despite considerable evolutionary distance.

Response: We thank the reviewer for this helpful suggestion. We have added a sentence in the Introduction noting that *C. neoformans*, *C. deneoformans*, and *C. gattii* complex species can form

hybrids despite considerable evolutionary divergence, with two additional references cited to provide this additional evolutionary context. The revised passage reads as follows (**lines 119–124**):

"Although these species are genetically distinct, hybrids have been documented, particularly between C. neoformans and C. deneoformans (referred to as AD hybrids, alluding to their different serotypes), and more rarely between C. neoformans and members of the C. gattii complex. However, these hybrids are typically diploid or aneuploid and exhibit reduced fertility and low spore germination rates (<20%), reflecting postzygotic barriers [14, 20, 21]."

Line 303 should site previous literature that identified rearrangements among the Cryptococcal species.

Response: We thank the reviewer for this helpful suggestion. We have now revised the paragraph (**lines 311–316**) to cite previous studies that identified chromosomal rearrangements among *Cryptococcus* species, including work comparing *C. neoformans*, *C. deneoformans*, and members of the *C. gattii* complex. We also cite Morrow et al. 2012 (mBio; PMID: 22375073) which documented the private translocation in strain H99.

Figure S5D the "pseudohyphae" and hyphae all look the same to me, and I would consider the pseudohyphae to look like true hyphae. Is there more rational for the differentiation beyond just degree of filamentation? In my experience the solo culture of DSM111204 is more typical of pseudohyphae.

Response: We thank the reviewer for this observation. The distinction between pseudohyphae and true hyphae was based primarily on microscopic morphology rather than degree of filamentation. Pseudohyphae (observed in NCYC1536 and DSM108351 × NCYC1536) were identified by constricted septa and irregular, chain-like elongation of yeast cells, whereas true hyphae (observed in DSM108352 × NCYC1536) were defined by continuous filamentous growth lacking constrictions and showing uniform hyphal morphology. This distinction has now been clarified in the legend of S5D Fig.

Figure 4C I would say those structures are much more characteristic of chlamydospores than basidia, particularly with the thickness of the cell wall, but difficult to tell from these images.

Response: We appreciate the reviewer's comment. We agree that we cannot presently exclude the possibility that the observed structures represent chlamydospores. In *C. neoformans*, chlamydospore-like structures have also been described (Lin & Heitman, 2005, Eukaryot. Cell; PMID: 15590819), although these typically display thicker and more refractile cell walls than those observed here. In contrast, the structures detected in *C. porticicola* more closely resemble *bona fide* basidia described in *Kwoniella* species (Statzell-Tallman et al., 2008; Metin et al., 2010; Guerreiro et al., 2013). However, given this uncertainty, we intentionally avoided making a definitive claim that they represent true sexual structures, referring to them instead as "basidia-like" or "putative sexual structures" throughout the text.

In response, we have revised the Results section to explicitly acknowledge that these structures could represent chlamydospores, which now reads in **lines 371–375**:

These structures emerged only after extended incubation (~1 month) and were not consistently observed across replicates. Although their identity could not be definitively confirmed and may represent chlamydospore-like cells, their morphology is more consistent with sexual development observed in related Kwoniella species [46-48].

And in the discussion section we now state in **lines 919–925**:

In mating assays, only one cross (DSM108352 × NCYC1536) produced hyphae and embedded basidia-like structures resembling those of Kwoniella rather than the typical aerial basidia of Cryptococcus, suggesting a potentially divergent mode of sexual development. Although these structures emerged only sporadically and their identity requires further ultrastructural or genetic confirmation, they appeared morphologically distinct from the chlamydospore-like cells reported in C. neoformans under stress or nutrient-limiting conditions, which are typically more darkly refractile and seem to possess thicker cell walls [80].

Figure 4E should have percent variation on axes.

Response: We thank the reviewer for this suggestion. The percentage of variance explained by each principal component was already indicated in the figure legend, but we have now also added these values directly to the axes of panel E (PC1 and PC2) to make the information immediately visible in the plot.

Figure 4G and corresponding results/discussion: Personally, I'm not convinced the four gamete test is appropriate for genome scale data. I would much prefer to compare to the LD50 values calculated in "The frequency of sex in fungi" by Nieuwenhuis & James. 13.9 kb would be halfway between S. cerevisiae and S. pombe and despite the authors claims in the discussion, is considerably higher than C. neoformans. As S. cerevisiae was estimated to undergo outcrossing at 1 in 10^5 generations and as S. pombe is thought to only undergo selfing (complicated by ancient admixture), this would suggest that mating (or at least outcrossing) in C. porticicola is exceptionally rare or only historic.

Response: We appreciate the reviewer's insightful critique regarding the use of the four-gamete test (FGT) for genome-wide analysis and thank the reviewer for stimulating this discussion. Upon careful consideration, we concur that the FGT has limitations when applied to genome-scale datasets. The method is based on the infinite-sites model of mutation, which assumes that each mutation occurs only once, and this condition is indeed often violated in practice. Recurrent or back mutations can therefore generate apparent four-gamete violations even in the absence of recombination, and the test itself does not provide a quantitative estimate of recombination rate. To avoid potential over-interpretation, we have removed the FGT analysis and the corresponding panel (previously Fig. 4G) from the manuscript.

We now focus exclusively on linkage disequilibrium (LD) decay as the primary genomic indicator of potential recombination, which is a more appropriate and widely used approach for genome-scale data. The LD50 value observed for *C. porticicola* (~13.9 kb), although somewhat higher than that reported for recombining *C. neoformans* subpopulations (5–7.5 kb; Desjardins et al., Genome Res. 2017; PMID: 28611159), remains within the same order of magnitude. It is also comparable to the LD50 reported for *Saccharomyces paradoxus* populations (~9 kb; Liti et al., Nature 2009; PMID: 19212322), lower than that of *Schizosaccharomyces pombe* (~19 kb), and substantially lower than *Candida albicans* (>100 kb), which is considered highly clonal. These values support

the interpretation that recombination occurs in *C. porticicola*, albeit possibly at lower effective frequency or within localized population contexts.

Accordingly, we have revised the corresponding paragraphs in the Results (**lines 390–397**) and Discussion (**lines 928–934**) sections to remove the FGT analysis, update Figure 4 panels and legend, and delete the associated methods description.

Some background on what centromeres look like in Cryptococcus could be helpful, as some readers may expect them to be point centromeres like in S. cerevisiae.

Response: Thanks for this helpful suggestion. To address this, we have expanded a sentence in the Results section (**lines 425–428**), which now reads:

In Cryptococcus, centromeres are regional rather than point-like, usually spanning tens of kilobases and characterized by a lack of coding sequences, enrichment in transposable elements—particularly long-terminal repeat (LTR) retrotransposons—and high levels of 5-methylcytosine (5mCG) DNA methylation [41, 45, 49-51]

The putative neocentromere is extremely interesting, but speculative at this point. This could just be a new TE island. It would be great to see follow up work on this given how closely related the strains with different karyotypes are.

Response: We thank the reviewer for raising this important point. We agree that the interpretation of this region as a potential neocentromere remains speculative at this stage. In response, we have toned down the language in the Results (**lines 430–445**), Discussion (**lines 865–875**), and Fig. 5 legend, describing this region more cautiously as a candidate centromere rather than a confirmed neocentromere. We now explicitly acknowledge that its centromeric function remains to be experimentally validated. We also added a statement in the revised Discussion (**lines 877–879**) to highlight future directions for testing centromere function.

The rapid change in 5S architecture is also very intriguing. In Schizosaccharomyces, 5S evolution is linked to meiotic drive and the selfish wtf genes. This could be interesting to include in the discussion, although no meiotic drive has ever been found in a basidiomycete to my knowledge.

Response: We thank the reviewer for drawing attention to the potential connection between 5S rDNA evolution and meiotic drive systems in some *Schizosaccharomyces* species. In response, we have added a new sentence to the Discussion (**lines 894–899**) citing De Carvalho et al. (eLife 2022; PMID: 36227631) to highlight that rapidly evolving *wtf* poison–antidote genes occur adjacent to dynamic 5S repeats and may exploit this repeat-rich environment for diversification. While no analogous system is known in *Cryptococcus*, this comparison provides valuable evolutionary context and raises the possibility that repeat-mediated mechanisms could contribute to the rapid 5S gene rearrangements we observe.

The section "Environmental detection of newly described species and limits of ITS-based detection" seems a bit tacked on. Some justification for why it is included would be nice or otherwise better integration to the rest of the manuscript.

Response: We thank the reviewer for this helpful comment. We agree that the section on environmental detection benefited from clearer integration into the overall manuscript. In response, we have moved this section (**lines 531–560**) to precede the formal taxonomic descriptions and revised its title and introductory paragraph to better convey its purpose, namely, to place the newly delineated *Cryptococcus* species in a broader ecological context by assessing their detectability in global fungal metabarcoding datasets. This analysis also provides insight into how effectively current ITS-based barcoding approaches distinguish closely related species.

Have all data underlying the figures and results presented in the manuscript been provided?

Large-scale datasets should be made available via a public repository as described in the *PLOS Genetics* data availability policy, and numerical data that underlies graphs or summary statistics should be provided in spreadsheet form as supporting information.

Reviewer #1: **No:** Seems like currently the sequencing datasets are still processing on NCBI. The BioProjects listed exist but there are no datasets in them.

Reviewer #2: Yes

We thank the reviewers for assessing this aspect. All numerical data underlying figures and summary statistics are provided as Supporting Information tables and files, and all scripts are available in the Zenodo repository cited in the manuscript. All sequencing datasets have been submitted to the NCBI Sequence Read Archive under the BioProjects listed in S6 Table and are now publicly released. The genome assemblies and annotations of *C. porticicola* DSM111204, DSM108352, and DSM111205 have also been submitted to NCBI; however, these records remain pending as they are undergoing manual processing, which has been delayed due to the current lapse in government funding. To ensure immediate accessibility, the assemblies and annotations are also available at Figshare (https://doi.org/10.6084/m9.figshare.30375169), which provides a permanent and citable record while GenBank processing is finalized.
